# The Loss of Masculine With Declined Serum DHT Is Associated With High Risk of Hepatocellular Carcinoma in Chinese Men

**DOI:** 10.3389/fendo.2020.00362

**Published:** 2020-06-30

**Authors:** Lichun Wang, Azhar Rasul, Zili Liu, Ying Pan, Weihua Wang, Jiang Li, Xiaomeng Li

**Affiliations:** ^1^The Key Laboratory of Molecular Epigenetic, Institute of Genetics and Cytology, Northeast Normal University, Changchun, China; ^2^Department of Zoology, Faculty of Life Sciences, Government College University Faisalabad, Faisalabad, Pakistan; ^3^Department of Urology Surgery, China-Japan Union Hospital of Jilin University, Changchun, China; ^4^Department of Prosthodontics, Dental Hospital of Jilin University, Changchun, China; ^5^Department of Prosthodontics, Affiliated Stomatology Hospital of Guangzhou Medical University, Guangzhou, China

**Keywords:** hepatocellular carcinoma (HCC), dihydrotestosterone, estradiol, liver function, liver injury

## Abstract

**Background:** Hepatocellular carcinoma (HCC) is a male-predominant cancer. However, the relationship between 5α-dihydrotestosterone (DHT), the active form of testosterone, and HCC risk has not been established yet.

**Methods:** We performed a serum epidemiological study in the Chinese population. From 2010 to 2012, 106 male HCC patients and 318 age-matched controls were detected for their serum DHT and estradiol (E2). The odds ratios (ORs) and 95% confidence interval (CI) were estimated by logistic regression analysis with adjustment for potential risk factors. Bivariate Pearson correlations between hormone concentrations and liver function index were investigated.

**Results:** Serum DHT levels were lower (to 1/3 of control), and E2 levels were higher (to 1.5-fold of control) in HCC patients. Compared with the low DHT level, men with a medium level had an adjusted multiple OR of 0.15 (95% CI 0.05–0.43, *p* trend < 0.01), and men with a high level had an OR of 0.05 (95% CI 0.01–0.21, *p* trend < 0.01). Notably, DHT concentration, but not E2, is correlated with liver injury.

**Conclusion:** The data suggest that serum DHT is closely associated with HCC risk, providing a reference in order to accurately predict liver cancer and study the pathogenesis of this disease.

## Introduction

Hepatocellular carcinoma (HCC) is one of the most common fatal cancers in the world (1–4). Notably, HCC is a significantly male-predominant cancer, with an overall sex ratio (m/f) around 2.4–1 (3, 5–8). Testosterone and estradiol (E2) have been suggested as determining such a preference (1, 9). Testosterone is metabolized into 5α-dihydrotestosterone (DHT) by 5α-reductase or converted into E2 by the aromatase in the liver, kidney, testes, and neural tissues (10). But the epidemiological studies show that the roles of testosterone for HCC risk are still controversial (11–16).

Although the serum E2 to testosterone ratio is reported higher in individuals with HCC (15), experimental data seem to suggest that normal hepatocytes apparently privilege 5α pathway, forming more biologically active androgens, DHT. HCC tissues appear much more active in metabolizing precursor testosterone in a privileged 5β metabolic pathway of androgens, followed with the decreased DHT level (17). Therefore, it seems more valuable to evaluate the HCC risk of DHT rather than testosterone.

Liver cancer incidence varies substantially among different races (1, 4, 17). The rate in Chinese men is about eight times higher than in US white men, and in Chinese-American men is ~3.5 times higher than local US white men (18, 19). Several studies show that the DHT/testosterone ratio is higher in whites than in Asian-Americans (20, 21). These findings suggest that the high incidence of HCC in Asian men may be related to the low level of DHT. However, to date, the association between the serum DHT level and the HCC risk in Asian men has not been fully addressed.

In the present work, focusing on Chinese men, we conducted a serum epidemiological case-control study to analyze the potential effects of serum DHT levels associated with HCC risk.

## Patients and Methods

### Subjects

We carried out a serum epidemiological study of HCC in Changchun, one of the biggest cities in the northeast of China. From 2010 to 2012, 106 HCC male cases diagnosed newly, aged 32 to 81, were selected from China-Japan Union Hospital, Jilin University. HCC was diagnosed on the basis of histological findings or an elevated level of serum α-fetoprotein more than 20 ng/ml combined with at least one positive image from angiography, sonography, and/or computed tomography. The 318 control subjects were randomly selected from healthy men who took part in regular health examinations in the same hospital. The controls were matched to 106 cases by age (±3 years) with a ratio of 3:1.

All the men received an initial baseline examination, including routine blood tests, urine tests, etiology analyses, conventional liver function tests, and an in-person interview conducted by trained research assistants using a structured questionnaire on demographic and anthropometric characteristics, lifetime habits of alcohol, and tobacco use, dietary pattern, as well as personal and family histories of major chronic diseases. A blood specimen also was collected.

### Laboratory Analyses

Serum samples were divided into 100-μl shares and frozen at −80°C until tested for hormonal measurements. Serum DHT and E2 were measured by enzyme-linked immunosorbent assay (ELISA). The DHT ELISA kits were purchased from DRG International USA, and the E2 ELISA kits were purchased from DRG Instruments GmbH, Germany (22–24). The main principle of these tests followed the typical competitive binding scenario. A set of standards was used to plot a standard curve from which the amounts of serum hormones in cases and controls were calculated. Paired cases and controls were assayed in the same run to avoid bias due to run-to-run variation.

### Statistical Analyses

Student *t*-tests were used to compare clinical and demographic features, which involved age, height, weight, body mass index (BMI), history of cigarettes, and alcohol, eight serum liver function indexes of albumin (ALB), globulin (GLB), total bilirubin (T-BIL), cholinesterase (CHE), alkaline phosphatase (ALP), alanine aminotransferase (ALT), aspartate aminotransferase (AST), and gamma-glutamyl transpeptidase (GGT), and serum hormone concentrations. Pearson correlations were calculated between hormone and liver function index to investigate their relationships to liver injury.

In the serum case-control study, subjects were divided into three groups for each sex hormone with cutoff for DHT of 500 and 1,255 pg/ml; for E2, 32.7 and 43.6 pg/ml. Odds ratios (ORs) and 95% confidence intervals (CIs) from binary logistic regression models, adjusted for age (<50, 50–59, 60–69, >70), BMI (<20, 20–25, 25–30, >30), smoking (never, previous, current), and alcohol consumption (never, previous, current), were used to assess serum hormone concentrations associated with the HCC risk.

In further studies, we deepened our research using three models. First, we divided all the participants into two parts by age (<65 or ≥65) and analyzed as the method above. Second, we divided the 106 cases by etiology and compared those with or without viral [hepatitis B virus (HBV) or hepatitis C virus (HCV)] infection to all the controls. Finally, we divided the 106 cases by alcohol/alcohol intake (ever or never) and compared them to the controls. ORs and 95% CIs from the binary logistic regression models were analyzed individually. All statistical analyses were calculated using SPSS 17.0 (SPSS, Chicago, IL, USA).

## Results

### Basic Demographic Features in the Subjects

The average age of subjects in this study was 62 years, and there was no significant difference in height, weight, or BMI (Table 1). Of the 106 liver cancer cases, 51 were infected with HBV or HCV, whereas the controls had none. Moreover, HCC cases had a higher ratio of alcohol consumption. Although they had almost the same drinking history of 10 years, the cases drank about twice as much (*p* < 0.01; 41.27 ± 76.191 vs. 19.77 ± 37.053 g per day), suggesting alcohol consumption as a risk factor for HCC.

**Table 1:** Demographic features of case and control.

**Variable**	**Mean ± SD**		***P*-value^$^**
	**Case (*n* = 106)**	**Control (*n* = 318)**	
Age	62.5 ± 9.1	62.4 ± 8.9	0.92
**Age range**			
31–40	2	5	
41–50	8	27	
51–60	34	101	
61–70	40	122	
71–80	22	63	
Height (cm)	171.5 ± 5.1	170.5 ± 5.1	0.26
Weight (kg)	67.9 ± 15.1	67.4 ± 11.2	0.86
BMI (kg/m^2^)^*^	23.1 ± 4.7	23.2 ± 3.5	0.89
**BMI range**			
<18.5	15	26	
18.5–24.0	57	171	
24.0–28.0	23	96	
>28.0	11	25	
**Alcohol history**			
g per day	41.27 ± 88.91	19.77 ± 37.05	**<0.01**
No. of years drunk	10.97 ± 18.26	10.39 ± 18.41	0.78
**Smoking history**			
No. of cigarettes per day	7.56 ± 11.18	6.18 ± 9.71	0.26
No. of years smoked	13.56 ± 16.76	14.58 ± 19.51	0.63
**Viral infection**			
HBV	51	0	
HCV	15	0	

* *BMI, body mass index [=weight (kg)/height (m)^2^]*;

$ *SD, standard deviation. Continuous variables were expressed as mean with standard deviation, and p-values were calculated by Student t-test*.

*HBV, hepatitis B virus; HCV, hepatitis C virus*.

*The Bold characters emphasize the statistical differences between the control group and the experimental group*.

### Significant Decline of Serum 5α-Dihydrotestosterone Levels in Hepatocellular Carcinoma Cases

**Figure 1:**
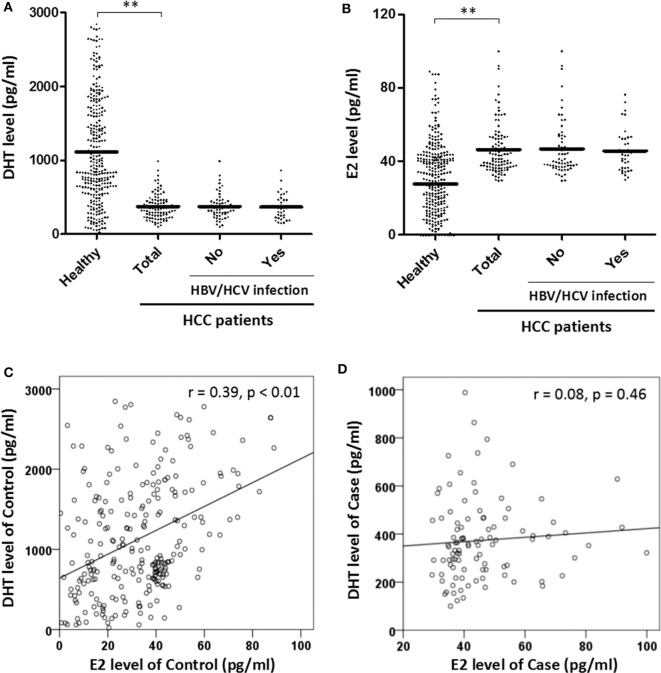
(1) Predicted mean concentrations (pg/ml) of sex hormones 5α-dihydrotestosterone (DHT) and estradiol (E2) in healthy and hepatocellular carcinoma (HCC) males by Student *t*-test **(A,B)**. HBV, hepatitis B virus; HCV, hepatitis C virus. (2) Assessed the relationship of DHT and E2 level in control and case groups by linear regression **(C,D)**. ^**^Represents that the *p*-value is < 0.01.

Since testosterone is the intermediate to both DHT and E2, it is better to measure directly their association to HCC. Using ELISA kits, we tested the serum DHT and E2 concentrations of all men (Figure 1). The results showed that DHT was two thirds lower in HCC cases (443.70 ± 326.788 vs. 1,293.89 ± 980.461 pg/ml, *p* < 0.01). Meanwhile, the E2 was significantly higher (47.79 ± 17.59 vs. 30.35 ± 33.71 pg/ml, *p* < 0.01). Further, a good linear trend of DHT and E2 was observed in healthy men (*r* = 0.39, *p* < 0.01) but disappeared in HCC patients, suggesting the unbalance.

### High Serum 5α-Dihydrotestosterone Is Related to the Decreased Hepatocellular Carcinoma Risk

In order to assess the HCC risk of DHT, ORs, 95% CIs, and *p*-values were calculated *via* dividing the patients into three groups by DHT level (≤ 500, 500–1,255, and ≥1,255 pg/ml) (Table 2). First, we estimated the crude OR and 95% CIs from binary logistic regression. The OR for those with DHT concentrations <500 pg/ml was referenced as 1.00. Those with a modest concentration between 500 and 1,255 pg/ml had an adjusted OR of 0.15 (95% CI 0.05–0.43), and those with the highest concentration, over 1,255 pg/ml, had an extreme OR of 0.05 (0.01–0.21). The *p* trend was < 0.01. The results showed that serum DHT levels were significantly negatively correlated with HCC risk.

**Table 2:** Multivariate-adjusted odds ratio (OR)^*^ and 95% confidence interval (CI) for HCC risk of DHT.

**Variable**	***n* (case/control)**	**OR (95% CI)**	***P*-value^#^**
**Total, pg/ml**			
≤ 500	82/59	1.00 (reference)	**<0.01**
500–1,255	20/121	0.15 (0.05–0.43)	
≥1,255	4/138	0.05 (0.01–0.21)	
**Age <65, pg/ml**			
≤ 500	51/29	1.00 (reference)	**<0.01**
500–1,255	13/78	0.06 (0.02–0.15)	
≥1,255	2/79	0.02 (0.01–0.07)	
**Age ≥65, pg/ml**			
≤ 500	31/30	1.00 (reference)	**<0.01**
500–1,255	7/43	0.19 (0.07–0.54)	
≥1,255	2/59	0.03 (0.01–0.16)	
**Smoking**			
No	64/173	0.11 (0.06–0.21)	**<0.01**
Yes	42/145	0.15 (0.08–0.29)	**<0.01**
**Alcohol history**			
No	79/235	0.13 (0.08–0.21)	**<0.01**
Yes	27/83	0.15 (0.07–0.33)	**<0.01**
**HCC classification**			
No HBV/HCV infection	65/318	0.11 (0.06–0.21)	**<0.01**
HBV/HCV infection	41/318	0.14 (0.07–0.28)	**<0.01**
**Prior BMI**			
Normal/Slim	25/247	0.15 (0.06–0.37)	**<0.01**
Overweight/Obese	3/21	0.35 (0.11–1.19)	0.09

* *Adjusted for BMI, age, smoking, alcohol intake*;

# *p-values were calculated by multivariate analysis, logistic regression model*.

*BMI, body mass index; DHT, 5α-dihydrotestosterone; HBV, hepatitis B virus; HCC, hepatocellular carcinoma; HCV, hepatitis C virus*.

*The Bold characters emphasize the statistical differences between the control group and the experimental group*.

By dividing the subjects by the median age of 65 years, the effect of age on the association between DHT and HCC risk was assessed. For the younger men, highest serum DHT concentration had lower multivariate ORs of 0.02 (95% CI 0.01–0.07) with a significant *p* trend. For the older men, the highest DHT levels had a similar multivariate 0.03 (95% CI 0.01–0.16) with *p* trend < 0.01. Thus, age does not play a pivotal role in the relationship between the low DHT level and the high HCC risk.

For virus infection factor, here, we showed that the OR values for the highest vs. lowest DHT concentration were 0.14 (95% CI 0.07–0.28, *p* < 0.01) in HCC patients with HBV or HCV infection and 0.11 (95% CI 0.06–0.21, *p* < 0.01) in HCC patients without viral infection. For lifestyle factors (smoking and alcohol intake), the OR values for the highest vs. lowest DHT concentration were 0.15 (95% CI 0.08–0.29, *p* trend < 0.01) in the smoking group and 0.11 (95% CI 0.06–0.21, *p* < 0.01) in the no smoking group; 0.15 (95% CI 0.07–0.33, *p* < 0.01) in the alcohol intake group and 0.13 (95% CI 0.08–0.21, *p* < 0.01) in the no alcohol intake group. These results demonstrated that the serum DHT is inversely associated with HCC risk, irrelevant to age, viral infection, smoking, or alcohol consumption. Interestingly, we found that the link between DHT and HCC risk seems to have disappeared in overweight males. However, the accurate evaluation between sex hormones and HCC risk among obese people needs a further confirmation based on the small sample size.

### High Serum Estradiol Is Related to the Increased Hepatocellular Carcinoma Risk

HCC cases exhibited significantly increased serum E2 levels. We thus investigated the significance of serum E2 for HCC risk. The E2 cutoff concentration was obtained by dividing the whole subjects into three groups: <32.7, 32.7–43.6, more than 43.6 pg/ml.

Table 3 presents the multivariate analysis of serum E2 as related to HCC risk. The OR for the low serum E2 concentration was referenced as 1.00. The modest group had a multivariate OR of 12.13 (95% CI 2.98–49.38), and the highest had an OR of 16.10 (95% CI 4.18–61.96). These data revealed the possible risk effect of high serum E2 on HCC incidence. This difference applied to different age ranged men, in viral infected (yes or no) men, and alcohol consumption or smoking (yes or no) men; all the *p* trends were < 0.01. Thus, the high E2 level is positively associated with HCC risk, but irrelevant to age, HBV, or HCV infection, alcohol consumption, and smoking.

**Table 3:** Multivariate-adjusted odds ratio (OR)^*^ and 95% confidence interval (CI) for HCC risk of E2.

**Variable**	***n* (case/control)**	**OR (95%CI)**	***P*-value^#^**
**Total, pg/ml**			
≤ 32.7	7/172	1.00 (reference)	**<0.01**
32.7–43.6	47/75	12.13 (2.98–49.38)	
≥43.6	52/71	16.10 (4.18–61.96)	
**Age <65, pg/ml**			
≤ 32.7	3/94	1.00 (reference)	**<0.01**
32.7–43.6	28/49	15.84 (4.28–58.68)	
≥43.6	35/43	25.19 (6.76–93.83)	
**Age ≥65, pg/ml**			
≤ 32.7	4/78	1.00 (reference)	**<0.01**
32.7–43.6	19/26	12.91 (3.67–45.49)	
≥43.6	17/28	11.32 (3.25–39.46)	
**Smoking history**			
No	64/173	3.46 (2.32–5.16)	**<0.01**
Yes	42/145	2.97 (1.84–4.83)	**<0.01**
**Alcohol history**			
No	79/235	2.91 (2.06–4.11)	**0.02**
Yes	27/83	4.20 (2.18–8.08)	**<0.01**
**HCC classification**			
No HBV/HCV^#^ infection	65/318	3.46 (2.26–5.32)	**<0.01**
HBV/HCV infection	41/318	3.44 (2.05–5.77)	**0.02**
**Prior BMI**			
Normal/Slim	25/247	3.60 (1.88–6.91)	**<0.01**
Overweight/Obese	3/21	6.57 (1.42–30.48)	**0.02**

* *Adjusted for BMI, age, smoking, alcohol intake*;

# *p-values were calculated by multivariate analysis, logistic regression model*;

# *HBV, Hepatitis B Virus*.

*BMI, body mass index; E2, estradiol; HCC, hepatocellular carcinoma; HCV, hepatitis C virus*.

*The Bold characters emphasize the statistical differences between the control group and the experimental group*.

### Declined Serum 5α-Dihydrotestosterone Significantly Correlated With Liver Injury

Next, we evaluated the association between serum hormones and eight commonly used indicators for assessing liver function. Compared with controls, in HCC cases, serum ALB and CHE were declined and GLB was increased (Table 4), implying the disorder of hepatic protein synthesis. Bilirubin (T-BIL) and four enzymes (ALP, ALT, AST, and GGT) were abnormally increased by about 2- to 5-fold, indicating the damage of liver parenchymal cells in HCC (25).

**Table 4:** Liver functional index in cases and controls.

	**Cases**	**Controls**	***P*-value**
ALB	34.99 ± 6.375	38.32 ± 6.114	**<0.001**
GLB	32.56 ± 8.059	25.60 ± 4.716	**<0.001**
CHE	4,965.30 ± 2,103.676	7,020.02 ± 1,810.776	**<0.001**
T-BIL	35.18 ± 52.109	16.64 ± 6.427	**0.001**
ALP	133.00 ± 119.050	64.83 ± 50.264	**<0.001**
ALT	56.57 ± 57.091	22.42 ± 12.839	**<0.001**
AST	73.03 ± 82.738	22.42 ± 12.839	**<0.001**
GGT	151.90 ± 229.510	29.16 ± 40.423	**<0.001**

*ALB, albumin; ALP, alkaline phosphatase; ALT, alanine aminotransferase; AST, aspartate aminotransferase; CHE, cholesteryl ester; GGT, γ-glutamyl transpeptidase; GLB, globulin; T-BIL, total bilirubin*.

*The Bold characters emphasize the statistical differences between the control group and the experimental group*.

Interestingly, almost all the liver function indexes showed a significant correlation with serum DHT (Table 5), rather than E2. The negative correlation of DHT with GLB (*r* = −0.297, *p* < 0.01) and positive correlation with CHE (*r* = 0.246, *p* < 0.01) suggest the potentially important role of DHT in hepatic synthetic function.

**Table 5:** Correlations between serum hormone and liver functional index in all the subjects.

	**Correlation in all subjects**	
	**DHT**	**E2**
ALB	0.132	−0.088
GLB	−0.297^**^	0.073
CHE	0.246^**^	−0.067
T-BIL	−0.147^*^	0.006
ALP	−0.200^**^	−0.006
ALT	−0.208^**^	0.118
AST	−0.225^**^	0.071
GGT	−0.199^**^	0.022

* *Significant correlated in 0.05 level (bilateral)*.

** *Significant correlated in 0.01 level (bilateral)*.

*ALB, albumin; ALP, alkaline phosphatase; ALT, alanine aminotransferase; AST, aspartate aminotransferase; CHE, cholesteryl ester; DHT, 5α-dihydrotestosterone; E2, estradiol; GGT, γ-glutamyl transpeptidase; GLB, globulin; T-BIL, total bilirubin*.

## Discussion

In this study, we performed a serum epidemiological case-control study setting Chinese men as subjects to assess the significance of sex hormones for HCC. The data displayed that the Chinese HCC males show extremely low serum DHT and higher E2 levels compared with the control group. The results of logistic regression analysis showed that the serum DHT and E2 were significantly negatively or positively correlated with HCC risk, respectively. For further study, we found that serum DHT rather than E2 is closely related to the liver injury.

HCC patients with cirrhosis have significantly lower plasma DHT concentrations than those with cirrhosis alone (26), and the serum DHT level appears lower in patients with HCC than in cirrhotic or normal men (27). This suggests that DHT may be a better marker of HCC than testosterone. Here, our study of Chinese men demonstrated the remarkable decline of serum DHT concentration in Chinese HCC cases (Figure 1A), consistent with previous reports. Moreover, we confirmed that the serum DHT level is negatively associated with HCC risk in this work (Table 2). Previous studies have demonstrated that the pathway of testosterone metabolism to DHT in liver cancer tissue is blocked (17), and our epidemiology also shows that the serum DHT concentration of HCC patients is lower. Granata et al. (17) pointed out that the metabolism of androsterone depends on 5β, rather than 5α reductase in HCC cells (17), which provides a reasonable explanation for the results. However, we need to further investigate the protein expression and activity of this enzyme to confirm the cause of the reduced DHT level.

Risk factors regarding liver cancer include alcoholism, chronic hepatitis, and exposure to compounds such as aflatoxin and aristolochic acid (AA) (28). Among them, the mutagens in the form of AA and its similar compounds exist in many traditional pharmacopeias in Asia. It has been found that HCC in the Asian population demonstrates unique mutation characteristics of AA exposure (29). *In vitro*, AA exposure induced hepatoma in a dose-dependent manner in mice, including HCC and intrahepatic cholangiocarcinoma (30). The evidence of epidemiological investigation indicates that the mutations induced by AA are possibly related to HBV (31, 32). In particular, Poon et al. (33) have identified AA-like mutations in 11 other non-HCC tumors as well, suggesting that the inappropriate employment of Chinese herbal medicine could increase the risk of cancer (not limited to HCC) in the Asian population.

Lifestyle and chronic HBV/HCV infection are another two important risk factors for HCC (34–36). Marrero et al. (36) have demonstrated that the risk of HCC increased 6-fold for drinking, 5-fold for smoking, and 4-fold with obesity in American patients. Our data show that Chinese HCC men have an ~2-fold increase with alcohol intake, less than that of Americans; while smoking has little effect on this disease in Chinese males (Table 1). The rate in Chinese men is about eight times higher than that in US white men (19), our findings thus suggest that the differences of HCC incidence among ethnic groups are closely related to lifestyle. Nevertheless, according to our study, the association of serum DHT or E2 with HCC risk does not seem to be related to these risk factors (Tables 2, 3) (e.g., drinking, smoking, and viral infection did not significantly increase or decrease the effect of hormones on HCC risk). This means that the risk factors of HCC such as lifestyle and viral infection may not affect hormone metabolism in Chinese men.

A higher increase in serum E2 levels was found in cases according to our study (Figure 1B), consistent with the previous report (14). But there was little significant correlation between E2 concentrations and liver function indexes (Table 5), indicating possible no association with liver injury, which was different from that of DHT. Patients with cirrhosis have lower DHT levels than healthy people, and DHT concentrations are inversely associated with liver injury according to MELD score (37). Our results support this assertion. Although previous studies provide evidence suggesting that androgen receptor can participate in HCC process in a hormone-independent manner (38). We speculate that increasing the DHT levels may separate androgen receptor from the signal pathway of liver injury.

Alpha fetoprotein (AFP) is a specific marker for the diagnosis of HCC, with a positive rate of about 70%, which is not sufficient to predict the occurrence of HCC. Since the incidence of HCC in men is higher than that in women, more studies have concentrated on the relationship between testosterone and the pathogenesis of HCC, but the results are contradictory (11–16). Previous studies have shown that the ratio of testosterone to E2 could be employed to predict HCC with cirrhosis (13), but the risk of liver cancer without cirrhosis has not yet been effectively evaluated. Our data revealed that the serum DHT level is negatively associated with HCC risk in this work (Table 2) and confirmed that the deviation factors such as virus exert a moderate effect on these results. This suggests that DHT could be a universal predictor of HCC, regardless of the cause of the disease.

In this study, we preliminarily determined the association of sex hormones (DHT and E2) with HCC risk *via* various statistical methods. Due to the small sample size, there is a certain degree of deviation in the strength of their correlation in the logistic stratified analysis. Especially in the analysis of the relationship between E2 and HCC, it is doubtful whether the OR and CI values obtained in this study are able to reflect the true correlation between them due to the limitation of sample size. In order to solve this problem, more HCC patients and a larger sample size are supposed to be included in the study. Therefore, for the further work, more observation objects will be collected to conduct a thorough and comprehensive investigation regarding the relationship between sex hormones (not limited to DHT and E2) and HCC risk.

## Conclusion

In this work, the DHT and E2 levels of 106 HCC patients and 318 age-matched controls were investigated among Chinese men. Compared with healthy people, HCC patients showed lower DHT and higher E2 concentrations, respectively. The results of logistic regression analysis suggested that both the serum DHT and E2 levels are closely related to HCC risk. Further studies indicated that DHT instead of E2 is associated with liver injury. Our study illustrates that serum DHT has the potential to be a better predictor of HCC compared to testosterone. For further work, we will expand the sample size to accurately determine the association of DHT with HCC risk. Moreover, the causal relationship between DHT and HCC will be explored through prospective experiments.

## Data Availability Statement

The datasets generated for this study are available on request to the corresponding author.

## Ethics Statement

The studies involving human participants were reviewed and approved by Animal Experiment Ethics Committee of Northeast Normal University. The patients/participants provided their written informed consent to participate in this study.

## Author Contributions

LW, ZL, AR, and XL contributed to conception and design. LW and ZL contributed to acquisition of data. LW and YP contributed to analysis and interpretation of data. LW and XL contributed to drafting of the manuscript. LW, WW, JL, and XL contributed to critical revision of the manuscript for important intellectual content. LW, ZL, and XL contributed to statistical analysis. JL and XL contributed to administrative support and study supervision. LW, AR, JL, and XL contributed to final approval of the manuscript. All authors contributed to the article and approved the submitted version.

## Conflict of Interest

The authors declare that the research was conducted in the absence of any commercial or financial relationships that could be construed as a potential conflict of interest.

## Abbreviations

HCC: hepatocellular cancer
DHT: 5α-dihydrotestosterone
E2: estradiol
AR: androgen receptor
ER: estrogen receptor
HBV: hepatitis B virus
HCV: hepatitis C virus
OR: odds ratio
CI: confidence interval
AFP: alpha fetoprotein
AFB1: aflatoxin B1
BMI: body mass Index
ALB: albumin
GLB: globulin
T-BIL: total bilirubin
CHE: cholesteryl ester
ALP: alkaline phosphatase
GPT: glutamic pyruvic transaminase
GOT: glutamic- oxalacetic transaminase
GGT: gamma-glutamyl transpeptidase
AA: aristolochic acid.
